# Glutathione Can Compensate for Salicylic Acid Deficiency in Tobacco to Maintain Resistance to *Tobacco Mosaic Virus*

**DOI:** 10.3389/fpls.2019.01115

**Published:** 2019-09-13

**Authors:** András Künstler, Lóránt Király, György Kátay, Alexander J Enyedi, Gábor Gullner

**Affiliations:** ^1^Plant Protection Institute, Centre for Agricultural Research, Hungarian Academy of Sciences, Budapest, Hungary; ^2^Office of Academic Affairs, Humboldt State University, Arcata, CA, United States

**Keywords:** glutathione, *NahG*, salicylic acid, tobacco, *Tobacco mosaic virus*, virus resistance

## Abstract

Earlier studies showed that the artificial elevation of endogenous glutathione (GSH) contents can markedly increase the resistance of plants against different viruses. On the other hand, salicylic acid (SA)-deficient NahG plants display enhanced susceptibility to viral infections. In the present study, the biochemical mechanisms underlying GSH-induced resistance were investigated in various tobacco biotypes displaying markedly different GSH and SA levels. The endogenous GSH levels of *Nicotiana tabacum* cv. Xanthi NN and *N. tabacum* cv. Xanthi NN *NahG* tobacco leaves were increased by infiltration of exogenous GSH or its synthetic precursor R-2-oxo-4-thiazolidine-carboxylic acid (OTC). Alternatively, we also used tobacco lines containing high GSH levels due to transgenes encoding critical enzymes for cysteine and GSH biosynthesis. We crossed Xanthi NN and NahG tobaccos with the GSH overproducer transgenic tobacco lines in order to obtain F_1_ progenies with increased levels of GSH and decreased levels of SA. We demonstrated that in SA-deficient NahG tobacco the elevation of *in planta* GSH and GSSG levels either by exogenous GSH or by crossing with glutathione overproducing plants confers enhanced resistance to *Tobacco mosaic virus* (TMV) manifested as both reduced symptoms (i.e. suppression of hypersensitive-type localized necrosis) and lower virus titers. The beneficial effects of elevated GSH on TMV resistance was markedly stronger in NahG than in Xanthi NN leaves. Infiltration of exogenous GSH and OTC or crossing with GSH overproducer tobacco lines resulted in a substantial rise of bound SA and to a lesser extent of free SA levels in tobacco, especially following TMV infection. Significant increases in expression of pathogenesis related (*NtPR-1a*, and *NtPRB-1b*), and glutathione S-transferase (*NtGSTtau*, and *NtGSTphi*) genes were evident in TMV-inoculated leaves in later stages of pathogenesis. However, the highest levels of defense gene expression were associated with SA-deficiency, rather than enhanced TMV resistance. In summary, elevated levels of glutathione in TMV-infected tobacco can compensate for SA deficiency to maintain virus resistance. Our results suggest that glutathione-induced redox changes are important components of antiviral signaling in tobacco.

## Introduction

The resistance of plants to virus infections is determined by the timely recognition of the invading virus by intracellular resistance (R) proteins (Padmanabhan and Dinesh-Kumar, 2014). Upon recognition, signals are transmitted to the nucleus leading to the rapid and extensive reprogramming of gene expression patterns in host plant cells. The reprogramming of the transcriptome is regulated by a complex, multilayered regulatory network, in which various transcription factors (Huang et al., 2017) and defense-related plant hormones (Alazem and Lin, 2015) play important roles. The defense hormone salicylic acid (SA, 2-hydroxybenzoic acid) plays a pivotal role in virus resistance of plants. Early studies showed that *Tobacco mosaic virus* (TMV) inoculation led to an early and marked accumulation of SA in the inoculated leaves of TMV-resistant *Nicotiana tabacum* cv. Xanthi NN. The highest levels of SA were detected in and around localized necrotic lesions (hypersensitive response, HR) that formed after TMV infection (Malamy et al., 1990; Enyedi et al., 1992). In the following years numerous investigations have confirmed the importance of SA in virus resistance (Gaffney et al., 1993; Delaney et al., 1994; Fodor et al., 1997; Singh et al., 2004; Lee et al., 2016; Klessig et al., 2018). In TMV-infected, resistant leaves most of the SA was found to be conjugated to glucose as SA-2-O-β-D-glucoside (SAG, also named as: O-β-D-glucosyl-SA) (Enyedi et al., 1992; Lee and Raskin, 1998). SA can be also converted into SA glucose ester (SGE), methyl salicylate 2-O-β-D-glucoside (MeSAG) and the volatile methyl salicylate (MeSA) in tobacco (Lee and Raskin, 1998; Dean et al., 2005; Vlot et al., 2009). SA-mediated signaling processes lead to the coordinate induction of genes encoding diverse pathogenesis-related (PR) proteins (Ward et al., 1991; van Loon et al., 2006) and ultimately to the development of systemic acquired resistance (SAR), which confers immunity to a broad spectrum of pathogens (Klessig et al., 2018). Although the exact roles of PR-1a and PR-1b proteins in virus resistance have remained elusive (Cutt et al., 1989; Linthorst et al., 1989), they are often used as markers for SA-mediated resistance in the early stages of viral pathogenesis (van Loon et al., 2006; Király et al., 2012; Gullner et al., 2017a; Klessig et al., 2018). Recent results revealed that PR-1 proteins possess sterol-binding activity, which hints to a novel antimicrobial function (Breen et al., 2017; Gamir et al., 2017).

When defense signal transduction is slow or inhibited the invading virus can replicate and spread systemically throughout the infected plants. The transgenic NahG tobacco line expresses a bacterial gene (*nahG*) encoding a salicylate hydroxylase enzyme that transforms SA into catechol (Friedrich et al., 1995). As a consequence, SA accumulation, disease resistance signaling and SAR are hampered in this transgenic line and therefore NahG tobacco is more susceptible to TMV infection than wild type plants (Gaffney et al., 1993; Király et al., 2002).

In resistant tobacco leaves TMV infection leads to oxidative stress i.e. the accumulation of reactive oxygen species (ROS) including superoxide and hydrogen peroxide, which results in lipid peroxidation and development of visible necrotic lesions (hypersensitive response, HR) (Doke and Ohashi, 1988; Király et al., 2008; Hernández et al., 2016). Infected plants deploy various antioxidative reactions in order to avoid excessive oxidative damage. The abundant cellular antioxidant tripeptide reduced glutathione (GSH, γ-L-glutamyl-L-cysteinyl-glycine) plays a principal role in the elimination of ROS *via* thiol-disulfide redox reactions (Noctor et al., 2012; Zechmann, 2014). Beyond its antioxidative role, GSH also acts as a signaling compound in infected plants (Ghanta and Chattopadhyay, 2011; Han et al., 2013; Gullner et al., 2017b). GSH and its oxidized disulfide form GSSG compose a redox buffer that contributes to the maintenance of cellular redox homeostasis (Schafer and Buettner, 2001). The redox state as a metabolic interface affects various biochemical processes including signaling and gene expression reprogramming associated with biotic stress responses (Foyer and Noctor, 2005; Potters et al., 2010). SA-mediated signaling also depends on the intracellular redox status. Notably, cellular SA levels are metabolically linked to those of hydrogen peroxide and GSH (Mateo et al., 2006; Han et al., 2013; Herrera-Vásquez et al., 2015; Gullner et al., 2017b). Thus the artificial elevation of foliar GSH contents either by an exogenous supply of GSH or S-nitrosoglutathione (GSNO) (Mateo et al., 2006; Kovacs et al., 2015), or by using transgenic plants overexpressing the GSH biosynthetic gene (*GSH1*) encoding γ-glutamyl-cysteine synthase (Ghanta et al., 2011, Ghanta et al., 2014) led to markedly increased SA levels as well as up-regulation of expression of the resistance marker gene *PR-1* and enhanced resistance to bacterial and fungal pathogens. On the other hand, treatments with exogenous SA resulted in increasing cellular GSH levels and elevated activities of glutathione reductase and glutathione S-transferase enzymes, along with enhanced virus resistance (Fodor et al., 1997; Srivastava and Dwivedi, 1998; Mateo et al., 2006). In infected plants SA induces defense gene expression *via* the redox-dependent activation of the essential transcription regulator protein NPR1. The inactive oligomer form of NPR1 is stabilized through intermolecular disulfide bonds in the cytosol. Upon SA accumulation the cellular redox potential becomes more negative by increasing GSH contents, which results in the reduction of NPR1 oligomers to a monomeric form. The monomeric, active form of NPR1 accumulates in the nucleus and activates defense gene expression (Mou et al., 2003).

Elevated GSH levels have often been observed in virus infected plant cells (Fodor et al., 1997; Király et al., 2002; Zechmann et al., 2005; Wang et al., 2011; Hernández et al., 2017). To test whether elevated endogenous GSH levels improve virus resistance, GSH levels were artificially elevated prior to virus inoculations. GSH levels can be substantially elevated by the synthetic cysteine precursor R-2-oxo-4-thiazolidine-carboxylic acid (OTC) in plant tissues (Hausladen and Kunert, 1990). OTC pretreatments of tobacco leaf discs prior to TMV inoculation indeed attenuated disease symptoms and also markedly decreased TMV coat protein levels (Gullner et al., 1999). Similarly, in *Zucchini yellow mosaic virus* (ZYMV) infected pumpkin plants OTC treatments markedly attenuated disease symptoms and reduced virus accumulation (Zechmann et al., 2007). In *Plum pox virus*-inoculated pea and peach tissues, OTC treatments also suppressed disease symptoms, although did not significantly reduce virus contents (Clemente-Moreno et al., 2010, Clemente-Moreno et al., 2012 and Clemente-Moreno et al., 2013).

In the present study, we have attempted to gain a deeper insight into the interaction of GSH and SA during defense responses to TMV infection in tobacco plants. We increased the cellular levels of GSH by using exogenous GSH and OTC treatments that resulted in elevated levels of SA in leaves of wild type Xanthi NN and SA-deficient NahG tobacco plants. In addition, we applied transgenic Burley NN tobacco lines that express genes encoding cysteine and GSH biosynthesis enzymes. These tobaccos overproduce GSH, as compared to wild type controls (Liszewska et al., 2001; Sirko et al., 2004; Wawrzyński et al., 2006). We crossed wild type Xanthi NN and SA-deficient NahG tobacco with these GSH overproducer plants in order to explore how different SA levels affect GSH-related defense reactions. TMV disease symptoms and virus multiplication rates were compared among different tobacco lines in order to better understand the biochemical mechanisms underlying TMV resistance. Our results revealed that elevated GSH levels can take over the signaling functions of SA and confer TMV resistance in SA-deficient tobacco lines comparable to that of wild type plants.

## Materials and Methods

### Plant Materials

The following tobacco cultivars and lines were used in our experiments: *Nicotiana tabacum* L. cv. Xanthi NN wild type and its SA-deficient line *N. tabacum* cv. Xanthi NN *NahG* (expressing the *nahG* transgene encoding salicylate hydroxylase) (Gaffney et al., 1993), *N. tabacum* cv. Burley NN wild type and its two glutathione overproducer transgenic lines *N. tabacum* cv. Burley NN *CEMK-9*, and *N. tabacum* cv. Burley NN *TRI-2*. The CEMK-9 transgenic tobacco line overexpresses two different transgenes encoding serine acetyltransferase (*EcSAT*) and *O*-acetylserine (thiol)-lyase (*EcOASTL*) (Liszewska et al., 2001), while the TRI-2 line overexpresses three transgenes encoding serine acetyltransferase *(EcSAT)*, γ-glutamylcysteine synthase (*EcGSH1*) and phytochelatin synthase (*SpPCS*) as described by Wawrzyński et al. (2006). *N. tabacum* cv. Burley and its transgenic lines are a kind gift of Dr. Agnieszka Sirko (Institute of Biochemistry and Biophysics, Warsaw, Polish Academy of Sciences). Tobacco plants were grown in the greenhouse with standard parameters (temperatures between 20–23°C, approximately 16 h of daylight with daily watering).

### Artificial Elevation of Glutathione Levels in Tobacco

In order to enhance glutathione contents in tobacco plants, we applied different methods. The first method involved increasing glutathione levels in SA-deficient NahG and wild type control Xanthi tobaccos by infiltrating intact leaf halves of plants with aqueous solutions of 2 mM reduced glutathione (GSH) or R-2-oxothiazolidine-4-carboxylic acid (OTC) (Sigma-Aldrich, USA) with a hypodermic syringe (Hafez et al., 2012). The corresponding leaf halves were infiltrated with tap water as a control. Third and fourth true leaves (counted from below) of 60–70 days old tobacco plants were infiltrated 2 days before TMV inoculation. In separate experiments, tobacco leaf discs were floated on the surface of aqueous solutions of OTC at different concentrations (Gullner et al., 1999). The third method we employed to increase GSH contents was crossing of SA-deficient NahG and wild type Xanthi tobaccos with glutathione overproducer tobacco lines (CEMK-9, TRI-2) by mechanical pollination (hand pollination) and using the F_1_ generation individuals for further experiments. To prove the success of crossings the presence of transgenes (*nahG*, *SAT*, *OASTL*, *GSH1*, and *PCS*) originating from different parents was verified in F_1_ generation individuals by polymerase chain reaction (PCR) with primers described earlier (*PpnahG*, Gaffney et al., 1993; *EcSAT*, Blaszczyk et al., 1999; *EcOASTL*, Liszewska et al., 2001; *EcGSH1* and *SpPCS*, Wawrzyński et al., 2006).

### TMV Inoculation and Evaluation of Disease Symptoms

*Tobacco mosaic virus* U1 strain (TMV) was maintained on a susceptible host, *Nicotiana tabacum* L. cv. Samsun nn. Systemically infected young upper leaves with strong mosaic symptoms were used for virus inoculations. TMV infected leaves homogenized in tap water (1 g of leaf in 10 ml of water) with silicon carbide (carborundum) powder as an abrasive were used to infect healthy leaves of 60–70 day-old tobacco by mechanical inoculation. Mock inoculations with tap water and carborundum powder were also performed as controls in experiments involving gene expression assays. TMV symptoms (HR-type localized necrotic lesions) were visually evaluated on locally infected (inoculated) tobacco leaves 5 days after virus inoculations when TMV symptoms are fully developed.

### Determination of Reduced (GSH) and Oxidized (GSSG) Glutathione

Plant tissue extraction and analyses of GSH and GSSG were carried out as described by Rellán-Álvarez et al. (2006) with some modifications. Leaf tissue samples (500 mg) taken from the third true leaves (counted from below) of 60–70 days-old-tobaccos were ground in liquid nitrogen. One biological sample represents 6 leaf halves (GSH or OTC treatment) or 6 leaves (crossing GSH overproducer and SA deficient plants) from 6 different plants. All assays were performed using three independent biological experiments with three technical replicates per biological sample. The tissue powder was suspended in four volumes of cold (4°C) extraction solution which contains 5% (w/v) stabilized metaphosporic acid (Acros Organics, USA) and 1 mM EDTA in 0.1% formic acid, supplemented with 1% (w/v) polyvinyl-polypyrrolidone just before use. The suspension was centrifuged at 14,000g for 15 min at 4°C. The supernatant was separated and the pellet was resuspended with two volumes of the same cold extraction solution, and centrifuged again. Both supernatants were combined and filtered through 0.45 μm Cromafil polyamide membrane filters (Macheray-Nagel, Germany). After HPLC separation and electronspray ionization mass spectrometric analyses (HPLC-ESI/MS) were performed with an HPLC MS system (Shimadzu, Japan) comprising two LC-20 AD high pressure liquid-delivery units, a SIL-20AC_HT_ autosampler, a CTO-20A column thermostat, an SPD-M20A UV/VIS photodiode array detector, and an LCMS 2020 single quadrupole mass spectrometer equipped with an ESI sample pulverization unit. The whole analytical system was guided by the LabSolution 5.72 software. Components of samples were separated on a Hypersil C18 packed column (200 mm × 4.6 mm, 5 μm particles) (MZ Analysentechnik, Germany) protected by means of a guard cartridge (10 mm × 4 mm, 5 μm particles). Separation of GSH and GSSG was achieved by means of a gradient elution at a constant flow rate of 0.65 ml min^−1^, the injected sample volume was 10 μl. Solvent A was 0.1% formic acid in ultrapure water whereas solvent B was 0.1% formic acid in acetonitrile. The applied gradient profile was as follows (minutes; B%): (0; 0%), (2.0; 10%), (9.0; 85%), (10.0; 85%), (10.5; 0%), (15.0; 0%). Samples were analyzed in negative ion mode. GSH was detected at m/z = 306, GSSG at m/z = 611. Unlabeled, analytical grade GSH and GSSG (Sigma-Aldrich, USA) were used as external standards for quantitation of GSH and GSSG levels in tobacco leaves.

### Determination of Free and Bound SA

The third true leaves (counted from below) of 60–70 days-old tobacco plants were analyzed to determine free and bound forms of SA essentially as described by Meuwly and Métraux (1993) using ortho-anisic acid (2-methoxybenzoic acid, oANI) as an internal standard. One gram of tobacco leaf tissue per sample was ground in liquid nitrogen and quartz sand. One biological sample represents 6 leaf halves (GSH or OTC treatment) or 6 leaves (crossing GSH overproducer and SA deficient plants) from 6 different plants. All assays were performed using three independent biological experiments with three technical replicates per biological sample. The grounded tissue was extracted with 70% methanol. Bound SA was hydrolyzed with 4 M hydrochloric acid (HCl). The following steps of the extraction were carried out as described in detail by Cole et al. (2004) and Pál et al. (2005). The separation of SA was performed by HPLC. Just prior to the HPLC analysis, the evaporated samples were resuspended in 500 µl of the HPLC starting mobile phase. SA was quantified fluorimetrically (W474 scanning fluorescence detector, Waters, USA), with excitation at 305 nm and emission at 407 nm.

### Analyses of TMV Accumulation and Tobacco Defense Gene Expression

To analyze TMV accumulation and tobacco defense gene expression 200 mg fresh plant leaf tissue per sample was collected and grounded in liquid nitrogen. Grounded leaf tissue was stored in an ultra-deep freezer at −70°C for several weeks until RNA isolation. One biological sample represents 6 leaf halves (GSH or OTC treatment) or 6 leaves (crossing GSH overproducer and SA deficient plants) from 3 different plants. The third and fourth true leaves (counted from below) were used from each plant. Total RNA contents (plant and virus) were isolated by the Plant Total RNA Extraction Miniprep System Kit according to manufacturer’s instructions (Viogene, USA). After RNA isolation DNAse I treatment with RQ1 RNase-Free DNase was performed (Promega, USA). RNA quantity and quality (260/280 and 260/230 ratio) was measured by a MaestroNano Spectrophotometer (Maestrogen, Taiwan) and RNA degradation was also checked by formaldehyde agarose gel electrophoresis of total RNA. One microgram total RNA was used for reverse transcription (RT) in each sample. RT was done with a RevertAid^™^ H^−^ cDNA Synthesis Kit (Thermo Fisher Scientific, USA) according to manufacturer’s instructions using both TMV coat protein gene reverse primer (*TMV-CP* rev) and an oligo-dT primer. An RT negative control (sample with RNA but without reverse transcriptase) was used during RT. Quantitative real-time PCR (qPCR) for assaying relative expression of TMV-CP and tobacco defense genes (*NtPR1-a*, *NtPRB-1b*, *NtGSTtau*, and *NtGSTphi*) was conducted with the 2×SYBR FAST Readymix reagent (KAPA Biosystems, USA). The qPCR reactions were conducted as described by Höller et al. (2010). In brief, the PCR reaction mix contained 7.5 µl KAPA SYBR FAST qPCR Master Mix (2X), 0.75 µl of 5 µM forward and reverse primers each, 3.5 µl PCR-grade water and 2.5 µl of 20-fold diluted cDNA in 15 µl total reaction volume. DNA amplifications were performed in a Bio-Rad CFX-96 real-time thermocycler (Bio-Rad, USA), running a standard program (95°C for 2 min, 40 cycles at 95°C for 10 s, 60°C for 10 s and 72°C for 30 s, followed by a melting curve analysis to determine amplicon specificity using a temperature range from 65 to 95°C with increments of 0.5°C). Gene expression was normalized to a tobacco *actin* gene (*NtAct*) as a reference. The suitability of *NtAct* as a reference gene was tested by analysis of cycle threshold (C_T_) variation in response to GSH/OTC treatments and TMV infection. Significant changes were not observed in C_T_ values (mean ± SD) for *NtAct* during treatments. All reactions were performed using three independent biological experiments with three technical replicates per biological sample. In each run, water-only controls and non-reverse-transcribed RNA were used as negative controls. The primer efficiencies for the genes tested were between 98–100%, therefore, changes in gene expression were calculated using the 2^−ΔΔCT^ method (Schmittgen and Livak, 2008). The following primers were used: tobacco actin gene (*NtAct*, GenBank accession X69885) 5’-CGGAATCCACGAGACTACATA-3’ (5’ primer) and 5’-GGGAAGCCAAGATAGAGC-3’ (3’ primer) amplicon size: 230 bp; TMV coat protein gene (*TMV-CP*, GenBank AJ429078) 5′-CTTGTCATCAGCGTGGGC-3′ (5′ primer) and 5′-AAGTCACTGTCAGGGAAC-3′ (3′ primer) amplicon size: 165 bp; tobacco pathogenesis-related gene 1a (*NtPR-1a*, GenBank D90196) 5′-GCAGATTGTAACCTCGTA-3′ (5′ primer) and 5′-CAATTAGTATGGACTTTCG-3′ (3′ primer) amplicon size: 297 bp; tobacco pathogenesis-related gene 1b (*NtPRB-1b*, GenBank X66942) 5′-GGGATACTCCACAACATTAG-3′ (5′ primer) and 5′-CACATACATATACACACC-3′ (3′ primer) amplicon size: 744 bp; tobacco tau class glutathione S-transferase gene (*NtGSTtau*, GenBank AY206006 and X56263) 5′-GATGGCAGAAGTGAAGTTG-3′ (5′ primer) and 5′-CTCCTAGCCAAAATSCCA-3′ (3′ primer) amplicon size: 488 bp; tobacco phi class glutathione S-transferase gene (*NtGSTphi*, GenBank AY206005) 5′-CTGGKGAWCACAAGAAGC-3′ (5′ primer) and 5′-GCCAARATATCAGCACACC-3′ (3′ primer) amplicon size: 490 bp.

## Results

### Induction of SA Accumulation in Tobacco Leaves by Infiltration of GSH and OTC

**Figure 1:**
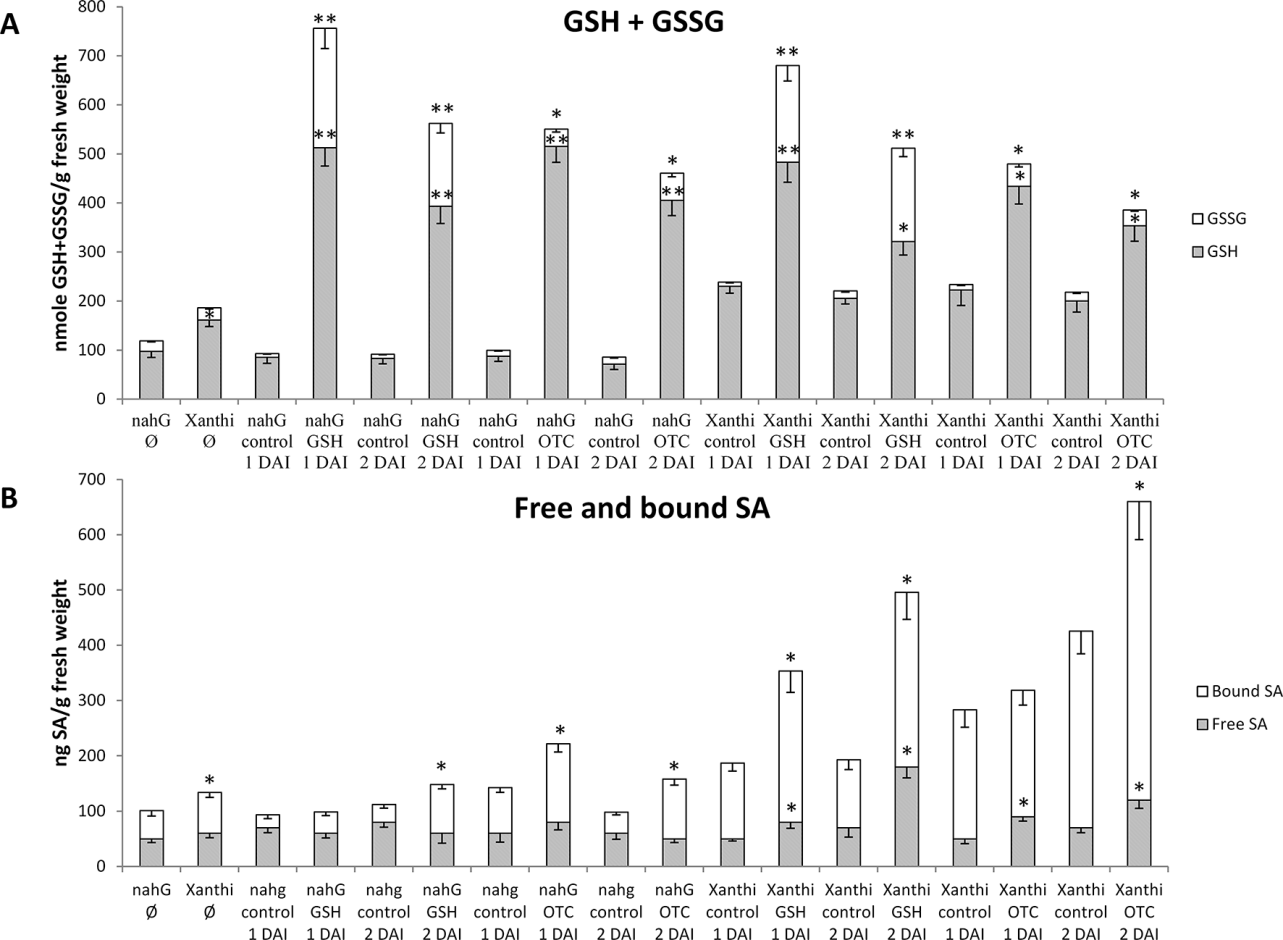
**(A)** Reduced (GSH) and oxidized (GSSG) glutathione levels and **(B)** free and bound salicylic acid (SA) levels in *Nicotiana tabacum* cv. Xanthi NN and its SA-deficient *NahG* line 1 and 2 days after infiltration (DAI) with 2 mM GSH or 2 mM R-2-oxothiazolidine-4-carboxylic acid (OTC). As a control, we infiltrated NahG and Xanthi plants with tap water. Ø = untreated tobacco. Asterisks (*, **) indicate statistically significant differences between GSH, GSSG, free and bound SA levels of control (water injected) and GSH or OTC treated leaf halves of the respective genotype at p ≤ 0.05 and p ≤ 0.01 (Student’s t-test). Columns represent means ± SD from three independent biological experiments. Asterisk (*) in case of untreated plants (Ø) indicates statistically significant differences (Student’s t-test, p = 0.05) between the NahG and Xanthi tobacco genotypes.

Our aim was to gain a deeper insight into the interaction of GSH and SA during defense responses to TMV infection in tobacco. First, we increased cellular levels of GSH in leaves of healthy, non-inoculated wild type Xanthi NN and SA-deficient NahG tobacco plants by exogenous GSH and OTC treatments and monitored changes in free and bound SA levels. Leaf halves were infiltrated with 2 mM aqueous solutions of GSH or OTC (control treatments: tap water). Both GSH and OTC treatments caused a substantial increase in total glutathione (GSH and GSSG) contents 1 and 2 days after treatments, regardless whether or not the plants accumulate wild type levels of SA. Interestingly, GSSG levels also increased significantly, especially following GSH treatments (**Figure 1A**). These results imply that elevation of *in planta* glutathione contents in tobacco may occur independently of SA.

In order to test the effect of elevated glutathione levels on *in planta* SA accumulation free and bound SA contents were assayed 1 and 2 days after GSH and OTC treatments. Wild type Xanthi NN tobacco contained significantly higher levels of bound SA, as compared to NahG plants and amounts of both free and bound SA in Xanthi NN increased 1.5 to 2-fold following GSH and OTC treatments, as compared to untreated controls. In SA-deficient NahG plants, GSH and OTC treatments did not increase free SA levels, however, bound SA levels were 1.5 to 2-fold higher in treated plants, as compared to untreated controls (**Figure 1B**). These results demonstrated that elevated levels of glutathione, following infiltration of exogenous GSH or OTC in tobacco leaves, markedly induced the accumulation of SA. To confirm these findings we tested the effect of elevated glutathione levels on *in planta* SA accumulation in a different experimental system: in this case tobacco leaf discs (diameter 1.5 cm) were continuously exposed to 2 mM or 5 mM OTC for 1, 2 or 3 days (**Figures S1, S2**). A 2 mM OTC treatment of wild type Xanthi NN tobacco leaf discs for 2 days resulted in the highest increase in SA levels, especially in the case of bound SA, as compared to the water-treated control (**Figure S1**). In SA-deficient NahG plants, the same OTC treatment did not increase the accumulation of free SA, however, bound SA levels were slightly but significantly higher in treated plants, as compared to controls (**Figure S2**). Our results demonstrated that in tobacco leaves, artificial elevation of glutathione levels may induce SA accumulation, either following a single infiltration of exogenous GSH or OTC or continuous exposure to OTC.

### OTC Induces Plant Defense Responses in Tobacco to a Similar Extent as SA

If OTC treatments induce *in planta* SA accumulation in tobacco, this implies that OTC can induce plant defense responses to a similar extent as SA. To test this hypothesis, we continuously exposed tobacco leaf discs to 2 mM OTC or 0.2 mM SA for 8, 24 and 48 h and assayed expression of the SA-mediated pathogenesis-related gene *NtPR-1a*. In wild type Xanthi NN tobacco *NtPR-1a* transcript levels were already induced in response to an 8 h exposure to SA but not OTC, while 24 and 48 h of both SA and OTC treatments resulted in elevated expression of *NtPR-1a*, especially in the case of SA exposure. In SA-deficient NahG plants, however, the induction of *NtPR-1a* transcripts was delayed as it was first detectable following a 24 h SA exposure, while 48 h of both SA and OTC treatments resulted in further elevated expression of *NtPR-1a* (**Figure S3**). Therefore, it seems that in tobacco, OTC can induce plant defense responses like *NtPR-1a* expression to a similar extent as SA, suggesting that OTC, possibly *via* GSH, can activate the same plant defense signaling pathway as SA.

### Artificial Elevation of Glutathione Levels by Infiltration of GSH and OTC Enhances Resistance to TMV in Wild Type and SA-Deficient NahG Tobaccos

**Figure 2:**
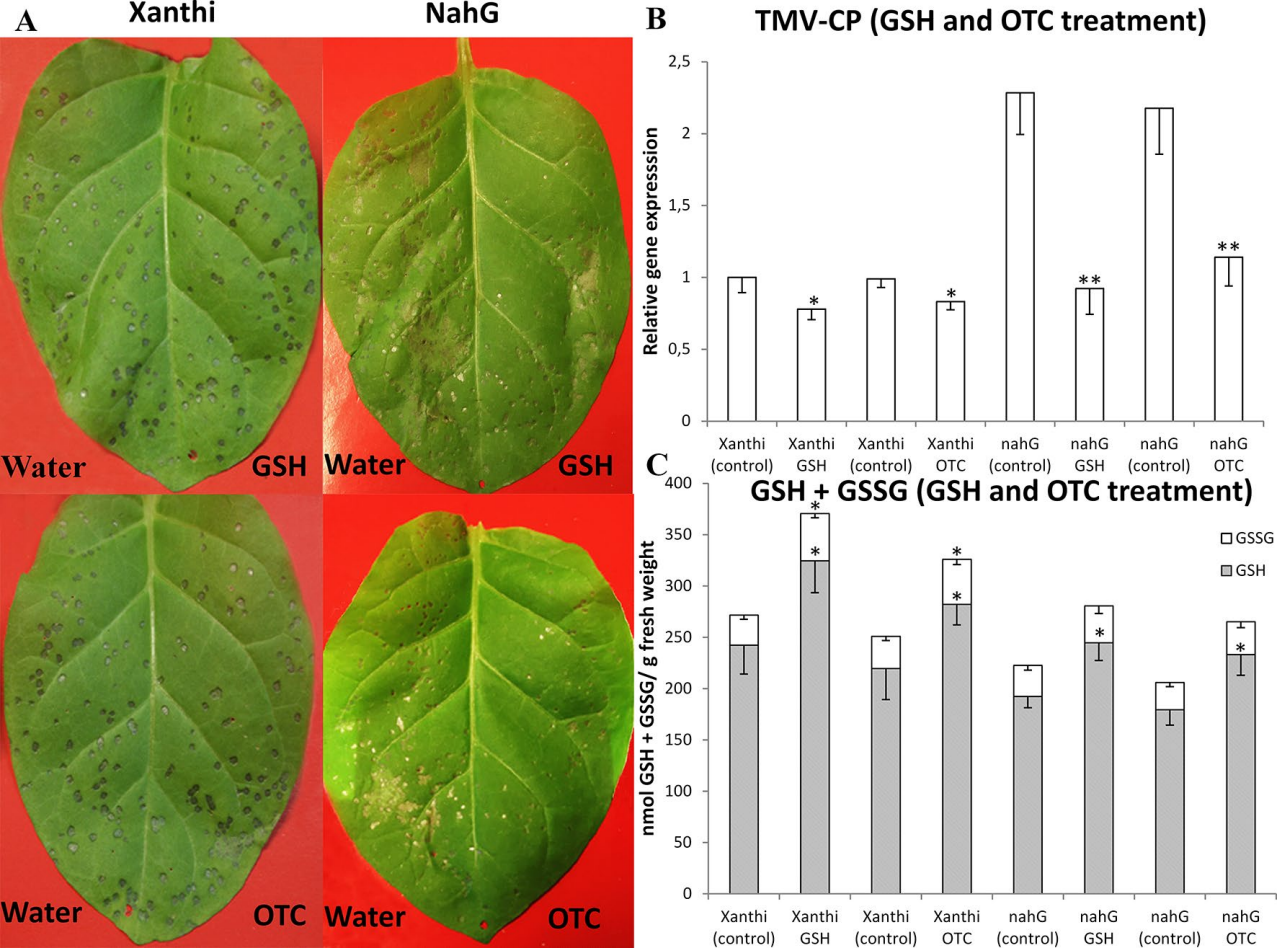
**(A)** Symptoms of *Tobacco mosaic virus* (TMV) inoculation, **(B)** relative expression of TMV coat protein gene (*TMV-CP*) and **(C)** levels of reduced (GSH) and oxidized (GSSG) glutathione in leaves of *Nicotiana tabacum* cv. Xanthi NN and its SA deficient *NahG* line 5 days after TMV inoculation. Right leaf halves infiltrated with 2 mM GSH (**A**, upper photos) or 2 mM R-2-oxothiazolidine-4-carboxylic acid (OTC) (**A**, lower photos). Left leaf halves infiltrated with pH 6.8 tap water as a control. GSH or OTC infiltrations were performed 2 days before TMV inoculations. Columns represent means ± SD from three independent biological experiments. Asterisks (*, **) indicate statistically significant differences in *TMV-CP* expression and GSH or GSSG levels of control (water injected) and GSH or OTC-treated leaf halves at p ≤ 0.05 and p ≤ 0.01 (Student’s t-test).

To directly assess the role of GSH in SA-mediated defense responses of tobacco to TMV infection, we first infiltrated 2 mM aqueous solutions of GSH or OTC into leaf halves of Xanthi and NahG tobaccos (control treatments: tap water). TMV inoculation was conducted 2 days after GSH and OTC infiltrations, since we found that in tobacco leaves, total glutathione and SA levels are significantly increased 2 days after a single infiltration of exogenous GSH or OTC (**Figure 1**). Visible symptoms, virus levels and accumulation of glutathione were evaluated 5 days after TMV inoculation when necrotic (HR) lesions are fully developed. In TMV-inoculated NahG leaves the expanding lesions coalesced, resulting in large necrotic patches indicating a deficiency in TMV resistance as described earlier (Gaffney et al., 1993; Delaney et al., 1994). However, GSH and OTC pretreatments caused a significant decline in necrotized leaf area and a more than 50% drop in TMV titers coupled to a more than 25% increase in levels of reduced glutathione (GSH) (**Figure 2**). Wild type Xanthi NN tobacco displayed localized, discrete necrotic (HR) lesions. Although GSH and OTC pretreatments did not cause a significant decline in necrotized leaf area they resulted in a ca. 20% drop in TMV titers, confirming earlier data that HR-type necrosis and virus resistance do not always correlate (Bendahmane et al., 1999; Künstler et al., 2016). Furthermore, GSH and OTC pretreatments of Xanthi NN tobacco caused a significant increase in GSH levels comparable to that in SA-deficient NahG plants (**Figure 2**). These results showed that the artificial elevation of glutathione levels in tobacco leaves may significantly increase TMV resistance and suggested that in SA-deficient NahG plants glutathione may restore wild type levels of TMV resistance.

### Glutathione Overproduction May Restore Resistance to TMV in SA-Deficient NahG Tobacco

**Figure 3:**
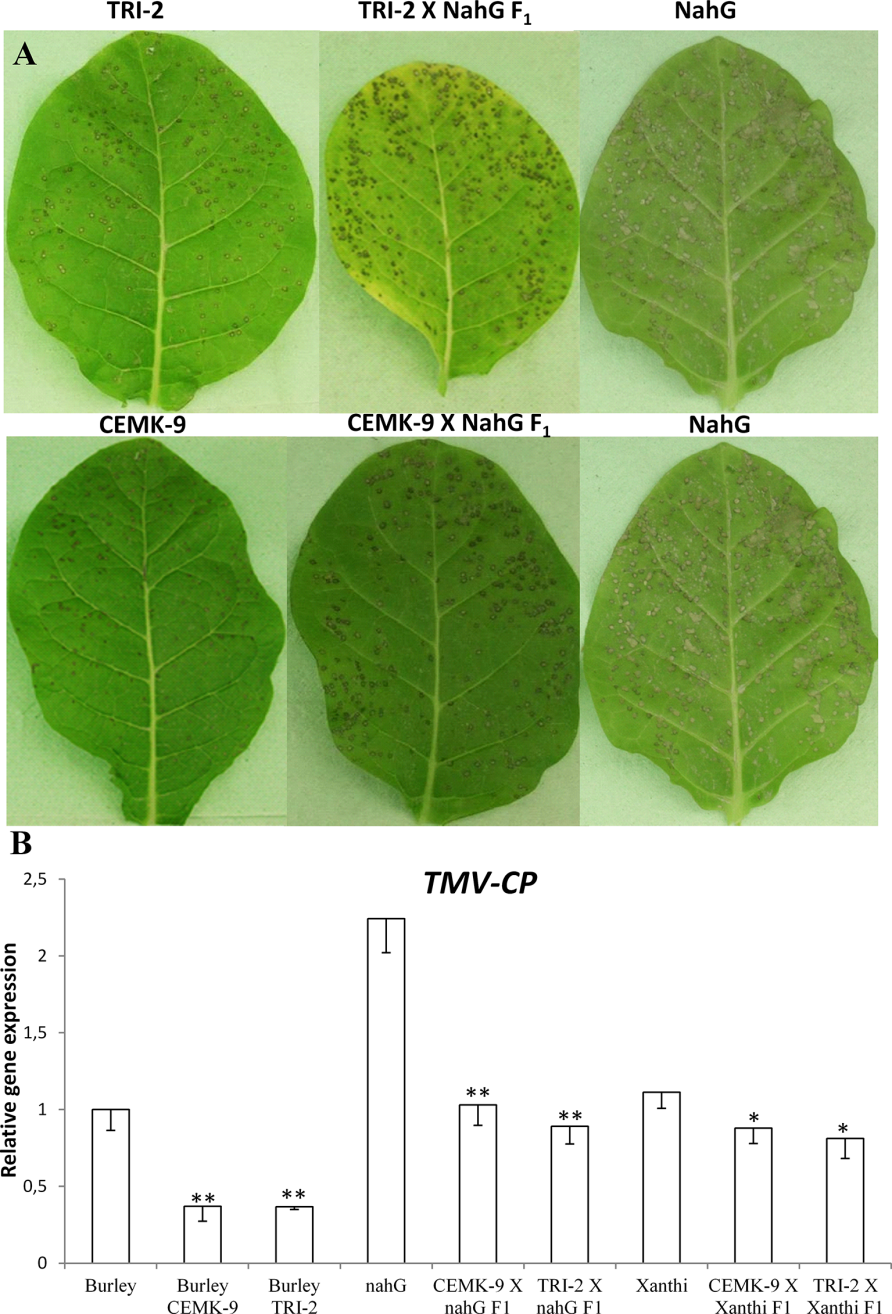
Disease symptoms of *Tobacco mosaic virus* (TMV) inoculation **(A)** and relative expression levels of TMV coat protein gene (*TMV-CP*) **(B)** in different tobacco lines 5 days after inoculation. Upper part **(A)** from left to right: *Nicotiana tabacum* cv. Burley NN *TRI-2* (glutathione overproducer), *N. tabacum* cv. Burley NN *TRI-2* X cv. Xanthi NN *NahG* F_1_ hybrid (glutathione overproducer X salicylic acid deficient), *N. tabacum* cv. Xanthi NN *NahG* (salicylic acid deficient). Lower part **(A)** from left to right: *N. tabacum* cv. Burley NN *CEMK-9* (glutathione overproducer), *N. tabacum* cv. Burley NN *CEMK-9* X cv. Xanthi NN *NahG* F_1_ hybrid (glutathione overproducer X salicylic acid deficient), *N. tabacum* cv. Xanthi NN *NahG* (salicylic acid deficient). Representative results of three independent biological experiments are shown. Relative expression levels of *TMV-CP* **(B)** as detected by real time RT-qPCR. The following tobacco lines were used: Burley NN and Xanthi NN (wild type plants); Burley NN CEMK-9 and TRI-2 (GSH overproducer lines); Xanthi NN NahG (SA-deficient line); CEMK-9 X NahG F_1_, TRI-2 X NahG F_1_, CEMK-9 X Xanthi F_1_ and TRI-2 X Xanthi F_1_ (F_1_ generation plants). Columns represent means ± SD from three independent biological experiments. Asterisks (*, p ≤ 0.05 and **, p ≤ 0.01) indicate statistically significant differences in *TMV-CP* expression by Student’s t-test between Burley – CEMK-9, Burley – TRI-2, as well as NahG – CEMK-9 X NahG F_1_, NahG – TRI-2 X NahG F_1_ and Xanthi – CEMK-9 X Xanthi F_1_, Xanthi – TRI-2 X Xanthi F_1_ in a pairwise manner.

In order to confirm that elevated levels of glutathione in tobacco enhance resistance to TMV and show that glutathione may indeed restore wild type levels of virus resistance in SA-deficient plants we made use of transgenic tobaccos that overproduce GSH (*N. tabacum* cv. Burley NN, CEMK-9, and TRI-2 lines). These plants overexpress genes encoding cysteine and GSH biosynthesis enzymes (serine acetyltransferase, SAT; O-acetyl-serine-(thiol)lyase, OASTL and γ-glutamylcysteine synthase, GSH1) (Liszewska et al., 2001; Sirko et al., 2004; Wawrzyński et al., 2006). We crossed SA-deficient NahG tobacco with these GSH overproducer plants. First we used glutathione overproducers as male (♂) and SA-deficient NahG and wild type Xanthi NN plants as female (♀) parents. Reciprocal crossings were also performed and the resulting F_1_ plants gave similar experimental results. In CEMK-9 X NahG F_1_ plants *EcSAT*, *EcOASTL*, and *PpnahG*, while in TRI-2 X NahG F_1_ hybrids *EcSAT*, *EcGSH1*, *SpPCS,* and *PpnahG* transgenes were detectable. In NahG parents only the *PpnahG* transgene was detectable (data not shown). F_1_ generation individuals were first evaluated for visible symptoms and virus levels 5 days after TMV inoculation when necrotic (HR) lesions are fully developed. Both parent lines that overproduce GSH (CEMK-9 and TRI-2) displayed localized, discrete HR-type lesions in inoculated leaves and a significantly smaller necrotized leaf area as compared to SA-deficient NahG tobacco (**Figure 3**). F_1_ generation hybrid plants originating from these parents (CEMK-9 and NahG or TRI-2 and NahG) showed an intermediate HR phenotype similar to that in wild type tobacco with normal GSH and SA levels. When virus accumulation was determined in the above mentioned lines we found that TMV titers are the highest in SA-deficient NahG tobacco and the lowest in GSH overproducer plants (CEMK-9 and TRI-2), while F_1_ hybrids from CEMK-9 X NahG and TRI-2 X NahG crosses display intermediate virus levels similar to those in wild type cv. Burley NN and Xanthi NN tobacco (**Figure 3**). Importantly, these results demonstrate that in SA-deficient NahG plants overproduction of glutathione may indeed restore wild type levels of TMV resistance. In fact, glutathione overproduction seems to significantly improve virus resistance (i.e. reduce TMV levels) regardless whether or not the plants accumulate wild type levels of SA (**Figure 3**).

### Defense Responses in F_1_ Hybrids of SA-Deficient and GSH Overproducer Tobaccos

**Figure 4:**
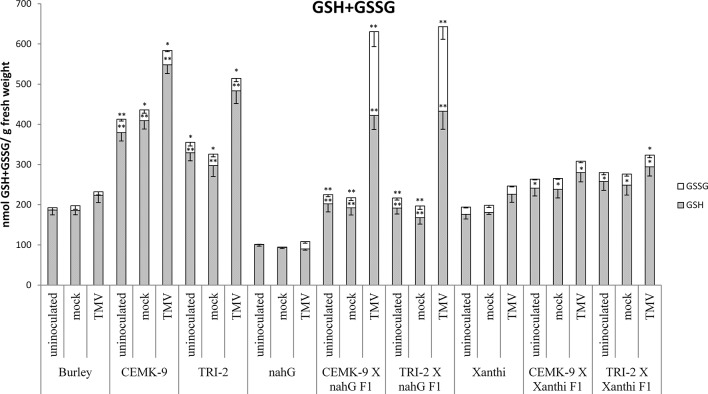
Reduced (GSH) and oxidized (GSSG) glutathione levels in uninoculated, mock-inoculated and *Tobacco mosaic virus* (TMV)-inoculated *Nicotiana tabacum* cultivars/lines. Detection of glutathione was performed 5 days after inoculation. For the description of tobacco lines see the legend of **Figure 3**. Columns represent means ± SD from three independent biological experiments. Asterisks (*, p ≤ 0.05 and **, p ≤ 0.01) indicate statistically significant differences by Student’s t-test in GSH or GSSG levels between Burley and its corresponding transgenic lines (CEMK-9 and TRI-2) as well as Xanthi and NahG versus their corresponding F_1_ hybrids in case of uninoculated, mock and TMV inoculated plants, respectively.

**Figure 5:**
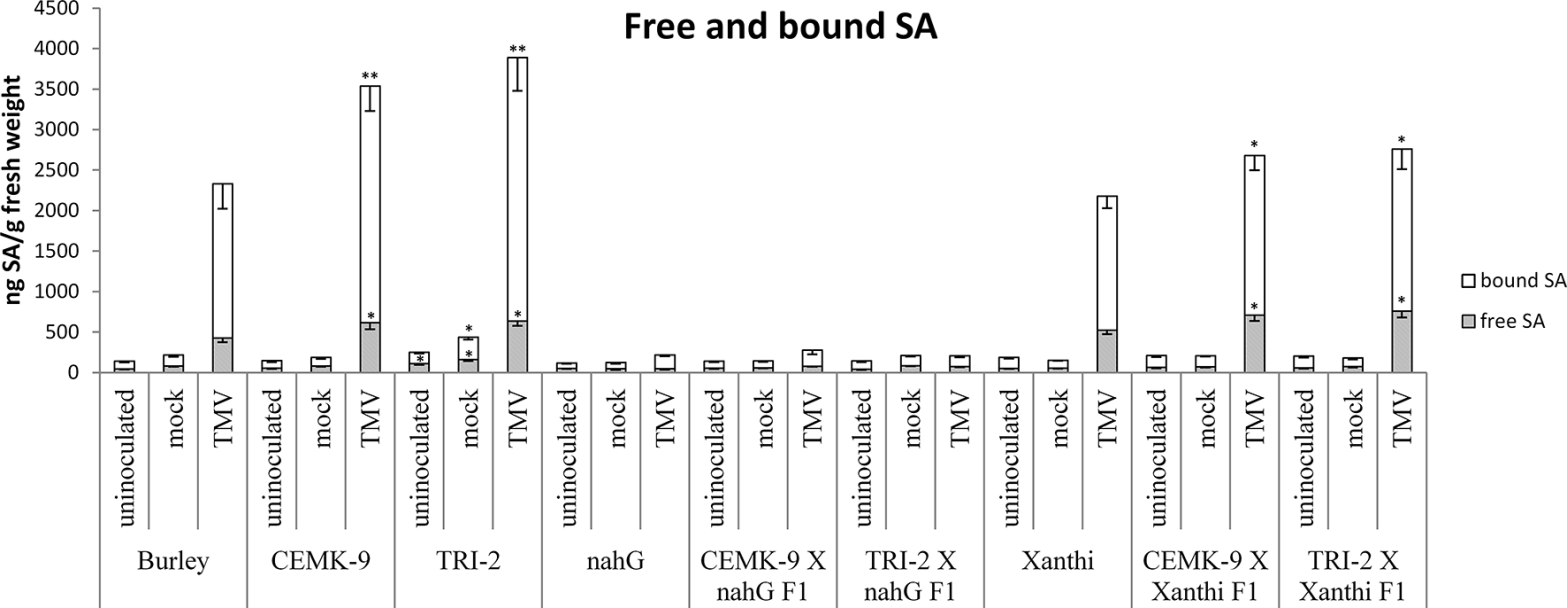
Free and bound salicylic acid (SA) levels in uninoculated, mock-inoculated and *Tobacco mosaic virus* (TMV)-inoculated *Nicotiana tabacum* cultivars/lines. Detection of SA was performed 5 days after inoculation. For the description of tobacco lines see the legend of **Figure 3**. Columns represent means ± SD from three independent biological experiments. Asterisks (*, p ≤ 0.05 and **, p ≤ 0.01) indicate statistically significant differences by Student’s t-test in free and bound SA levels between cv. Burley and corresponding transgenic lines (CEMK-9 and TRI-2) as well as Xanthi and NahG versus their corresponding F_1_ hybrids in case of uninoculated, mock and TMV inoculated plants, respectively.

**Figure 6:**
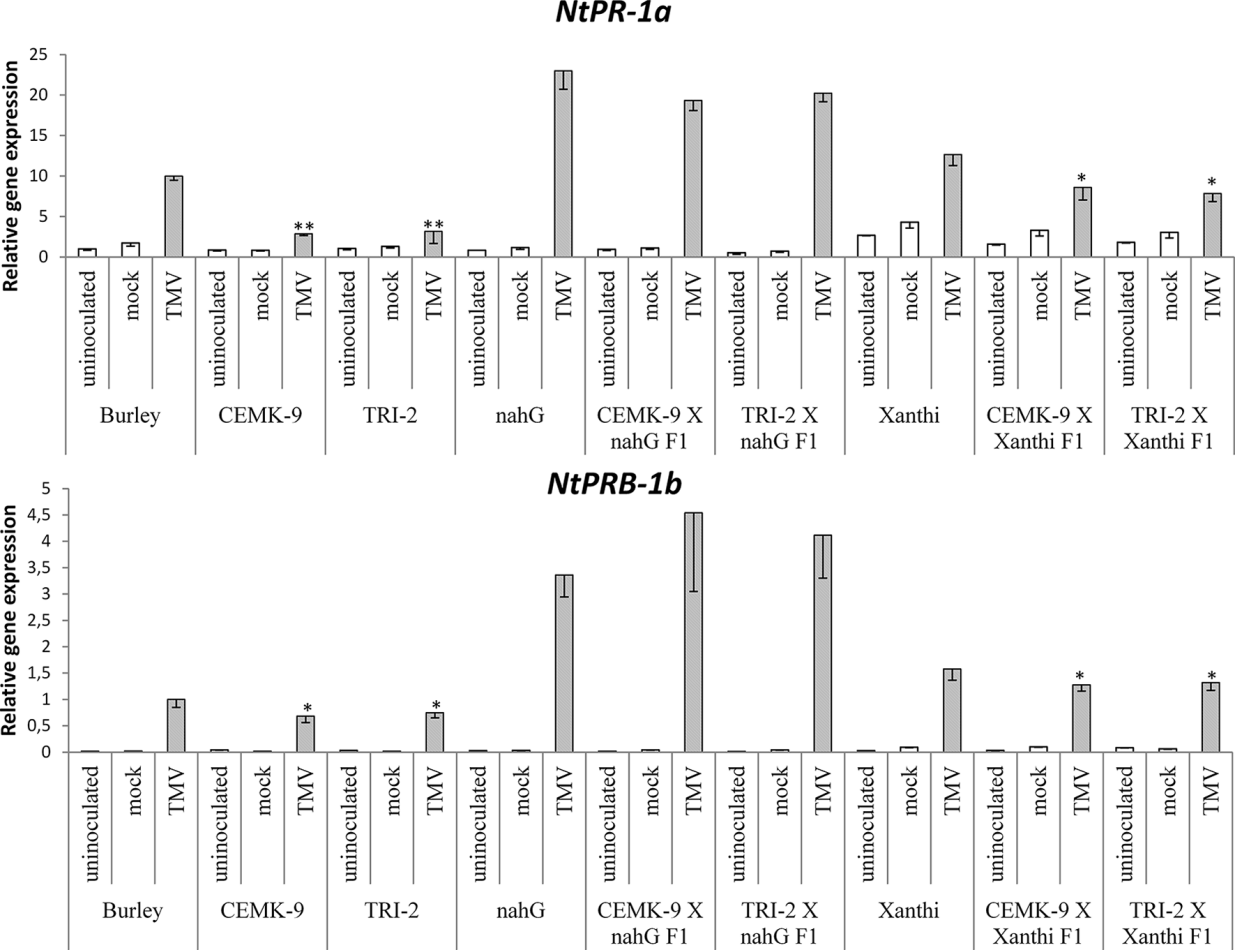
Expression levels of two pathogenesis related genes (*NtPR-1a* and *NtPRB-1b*) in uninoculated, mock-inoculated and *Tobacco mosaic virus* (TMV)-inoculated *Nicotiana tabacum* cultivars/lines. Analyses of gene expression were performed 5 days after TMV-inoculation by real time RT-qPCR. For the description of tobacco lines see the legend of **Figure 3**. Columns represent means ± SD from three independent biological experiments. Asterisks (*, p ≤ 0.05 and **, p ≤ 0.01 ) indicate statistically significant differences (Student’s t-test) in gene expression between cv. Burley and corresponding transgenic lines (CEMK-9 and TRI-2) and cv. Xanthi NahG and cv. Xanthi versus their corresponding F1 hybrids in case of uninoculated, mock and TMV inoculated plants, respectively.

**Figure 7:**
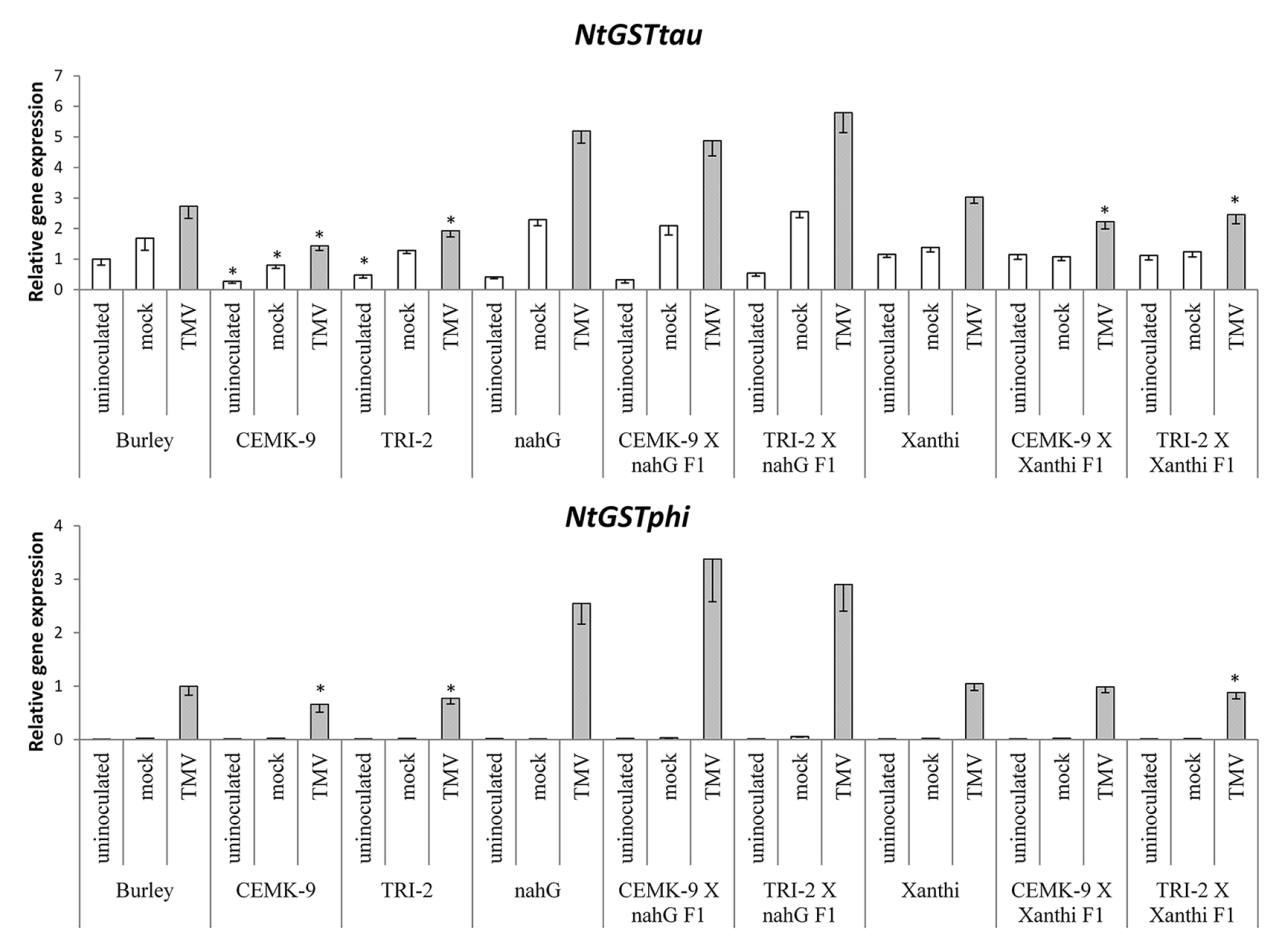
Expression levels of two glutathione S-transferase (GST) genes (*NtGSTtau* and *NtGSTphi*) in uninoculated, mock-inoculated, and *Tobacco mosaic virus* (TMV)-inoculated *Nicotiana tabacum* cultivars/lines. Detection of GST gene expression was performed 5 days after TMV-inoculation by real time RT-qPCR. For the description of tobacco lines see the legend of **Figure 3**. Columns represent means ± SD from three independent biological experiments. Asterisks (*, p ≤ 0.05 ) indicate statistically significant differences (Student’s t-test) in gene expression between cv. Burley and corresponding transgenic lines (CEMK-9 and TRI-2) and cv. Xanthi NahG and cv. Xanthi versus their corresponding F_1_ hybrids in case of uninoculated, mock and TMV inoculated plants, respectively.

We also used F_1_ hybrids from crosses between SA-deficient and GSH overproducer tobacco lines to explore how different endogenous SA levels affect *in planta* GSH accumulation and GSH-related defense reactions in response to TMV infection. First we assessed levels of reduced (GSH) and oxidized (GSSG) glutathione in uninoculated, control inoculated (mock), and TMV-inoculated leaves of these tobaccos, 5 days after inoculations (**Figure 4**). Interestingly, high levels of total glutathione seem to be clearly associated with a higher resistance to TMV (compare **Figures 3**, **4**). However, glutathione levels were extraordinarily high in TMV-inoculated F_1_ hybrids from CEMK-9 X NahG and TRI-2 X NahG crosses, primarily due to a several fold increase in levels of GSSG, as compared to controls (**Figure 4**). This could indicate the pivotal role of glutathione in restoring TMV resistance in the background of SA deficiency, including the suppression of oxidative stress (HR-type necrotic lesions) in virus-infected tissues that could result in GSSG accumulation. An analysis of free and bound SA levels in the above mentioned tobacco lines revealed a significant increase evident in TMV-inoculated leaves as compared to controls (**Figure 5**). Furthermore, high levels of free and bound SA seem to be clearly associated with a higher resistance to TMV, in a similar manner as glutathione (compare **Figures 3**–**5**), except for SA-deficient CEMK-9 X NahG and TRI-2 X NahG F_1_ plants. Assessing expression of SA-mediated and GSH-associated defense gene expression we monitored changes in transcript levels of two pathogenesis-related and two glutathione S-transferase genes (*NtPR-1a*, *NtPRB-1b*, *NtGSTtau*, and *NtGSTphi*) 5 days after virus inoculations. A significant increase in gene expression was evident in TMV-inoculated leaves as compared to controls. However, the highest levels of defense gene expression seem to be associated with SA-deficiency and a decreased GSH/GSSG ratio, rather than enhanced TMV resistance (compare **Figures 6** and **7** to **Figures 3**–**5**). This could indicate higher than normal levels of oxidative stress in virus-infected SA-deficient tobacco cells in later stages of pathogenesis (5 days after inoculation, DAI) when necrotic (HR) lesions are fully developed, even if glutathione accumulation and TMV resistance is comparable to that of wild type plants. Furthermore, the induction of these defense-related genes could possibly aid in preventing an opportunistic fungal or bacterial infection.

## Discussion

Our results showed that GSH and SA may induce partially overlapping signaling pathways in tobacco to confer resistance to TMV. Exogenous treatments with GSH or the synthetic cysteine precursor OTC caused a substantial increase in total glutathione (reduced GSH and oxidized GSSG), regardless whether or not the plants accumulate wild type levels of SA (**Figure 1A**). This indicates that elevation of *in planta* glutathione contents in tobacco could occur independently of SA. In fact, it has been demonstrated that cellular GSH levels may determine *in planta* SA contents. Artificial elevation of foliar GSH either by exogenous treatments (Mateo et al., 2006; Kovacs et al., 2015), or using transgenic plants overexpressing the GSH biosynthetic gene encoding γ-glutamylcysteine synthase (Ghanta et al., 2011) led to markedly increased SA levels both in tobacco and *Arabidopsis thaliana*. Accordingly, we found that artificial elevation of glutathione levels may induce SA accumulation in tobacco leaves, either following a single infiltration of exogenous GSH or OTC or continuous exposure to OTC (**Figure 1B**, **Figures S1** and **S2**) Furthermore, although GSH and OTC treatments of SA-deficient NahG tobacco did not increase free SA levels, bound SA levels were unexpectedly (1.5 to 2-fold) higher in treated NahG plants. This could be due to the suppressed antioxidant capacity of NahG tobacco including glutathione levels 50% of wild type (Király et al., 2002; **Figures 1A** and **4**). Thus, an influx of exogenous GSH or OTC is followed by a dramatic elevation of endogenous GSH and GSSG in NahG tobacco, as compared to wild type (**Figure 1A**). Elevated glutathione levels could signal the accumulation of bound SA in a similar manner as found in glutathione overproducing transgenic tobaccos that contain ca. twice as high levels of GSH, GSSG, and bound SA as wild type plants (Matern et al., 2015). It is plausible that the accumulation of bound SA in GSH or OTC treated NahG tobacco is a means of “saving” SA by escaping the salicylate hydroxylase activity encoded by *nahG*. In TMV-infected, resistant tobacco most of the SA is conjugated (bound) to glucose as SAG, SGE and MeSAG (Enyedi et al., 1992; Enyedi and Raskin, 1993; Lee and Raskin, 1998; Vlot et al., 2009). The biological relevance of SA-glucosides is not known, but they can be rapidly converted into free SA, therefore, could play a role in plant disease resistance (Hennig et al., 1993; Yao et al., 2007; Pastor et al., 2013).

We demonstrated that OTC treatments induce *in planta* SA accumulation in tobacco, which implies that OTC induces plant defense responses to a similar extent as SA. Indeed, the rise of endogenous GSH leads to elevated SA levels (Mateo et al., 2006; Herrera-Vásquez et al., 2015) and up-regulated expression of the resistance marker gene *PR-1a* in foliar tissues (Gomez et al., 2004; Ghanta et al., 2011). The relationship between endogenous GSH and *PR-1* expression was also observed in a *lesions simulating disease* (*lsd*) mutant of *Arabidopsis thaliana* that spontaneously develops HR-like necrotic lesions (Senda and Ogawa, 2004). Indeed, our results proved that OTC treatments (continuous exposure up to 48 h) lead to a substantial and early up-regulation of *PR-1a* expression to a similar extent as SA, in both Xanthi NN and SA-deficient NahG leaves, although gene induction is slower and less pronounced in NahG plants (**Figure S3**). This is in line with earlier findings that up-regulation of *PR-1a* is a reliable marker of the SA mediated defense pathway (Malamy et al., 1990, Ward et al., 1991; Delaney et al., 1994).

Earlier studies showed that the artificial elevation of endogenous GSH levels in virus-infected plant leaves by the application of the synthetic cysteine precursor OTC results in strongly attenuated disease symptoms and often also in decreased virus contents (Gullner et al., 1999; Zechmann et al., 2007; Clemente-Moreno et al., 2010; Wang et al., 2011; Clemente-Moreno et al., 2012 and Clemente-Moreno et al., 2013). Nevertheless, the exact biochemical mechanisms underlying GSH-elicited virus resistance are still elusive. In the present study we compared the antiviral effects of elevated foliar GSH contents between TMV-inoculated wild-type Xanthi NN tobacco and transgenic NahG tobacco, in which the accumulation of SA is prevented and thus SA-mediated antiviral defense is impaired (Gaffney et al., 1993; Friedrich et al., 1995). As the cellular levels of SA and GSH are physiologically coupled by unknown mechanisms (Mateo et al., 2006; Herrera-Vásquez et al., 2015), we expected that infiltration of leaves with GSH or OTC would exert different effects on TMV resistance in SA-deficient NahG tobacco than in wild type Xanthi. Following TMV-inoculation, the number and size of HR-type necrotic lesions were significantly larger in NahG as compared to Xanthi confirming earlier results (Gaffney et al., 1993; Delaney et al., 1994; Király et al., 2002) and indicating enhanced TMV spread and replication (**Figures 2**, **3**). In *Cucumber mosaic virus* or in *Turnip crinkle virus*-infected *A. thaliana* leaves, virus replication levels were also higher in an SA-deficient NahG biotype than in wild type Col-0 plants during the first 10 days of infection, while SA-deficient plants displayed lower virus titers than the wild-type seedlings at 20 and 25 days post-inoculation, indicating a possible adaptation of SA-deficient plants to virus infections in later stages of pathogenesis (Wang et al., 2011). Intriguingly, in our experiments GSH and OTC treatments led to a much more robust reduction of virus titers in NahG than in wild type Xanthi (**Figure 2**), perhaps reflecting a similar adaptation process. Importantly, our results showed that artificial elevation of GSH levels significantly increase TMV resistance and suggested that GSH can restore wild type levels of TMV resistance in SA-deficient NahG plants. Previous research has shown that enhanced TMV resistance (significantly reduced HR and virus titers) of cv. Samsun NN tobacco with sufficient sulfate supply was accompanied by enhanced levels of GSH and its precursor cysteine (Király et al., 2012), while in genetically susceptible tobacco (Samsun nn) a sufficient sulfate supply induced TMV resistance (delayed mosaic symptoms and reduced TMV titers) also associated with GSH and cysteine accumulation (Höller et al., 2010). In fact, cysteine is also required for plant resistance to e.g. bacterial infections (Alvarez et al., 2012). Recently, De et al. (2018) reported that inhibition of GSH accumulation by silencing of *GSH1* during *Potato virus X* (PVX) infection enhances virus replication. Importantly, these findings point to GSH and its precursor cysteine as not only antioxidants but also as signaling agents of plant virus resistance.

To corroborate the above findings on effects of elevated GSH on virus resistance in tobacco, we applied a different experimental approach by using two transgenic lines that overexpress genes encoding critical enzymes for cysteine and GSH biosynthesis. *N. tabacum* cv. Burley CEMK-9 overexpresses a *SAT* and *OASTL* gene, whereas Burley TRI-2 harbors extra copies of *SAT*, *GSH1*, and *PCS* genes. SAT and OASTL catalyze the last steps of cysteine biosynthesis, whereas GSH1 is the rate-limiting enzyme of GSH biosynthesis, and PCS is involved in phytochelatin biosynthesis (Liszewska et al., 2001; Sirko et al., 2004; Wawrzyński et al., 2006). We crossed the GSH overproducer CEMK-9 and TRI-2 lines with Xanthi NN and SA-deficient NahG tobaccos in order to obtain F_1_ hybrids (CEMK-9 X Xanthi, TRI-2 X Xanthi, CEMK-9 X NahG and TRI-2 X NahG). All plants displayed localized necrotic lesions (HR) in TMV-inoculated leaves, the extent of HR being most severe in NahG tobacco, while GSH-overproducing CEMK-9 and TRI-2 lines displayed the weakest HR. Interestingly, CEMK-9 X NahG and TRI-2 X NahG F_1_ plants showed an intermediate HR phenotype similar to that in wild type tobacco and TMV titers correlated well with the extent of HR-type necrosis (**Figure 3**). Importantly, virus titers in CEMK-9 X NahG and TRI-2 X NahG F_1_ hybrids were substantially lower than in NahG plants, clearly demonstrating that elevation of endogenous GSH levels by genetic tools (i.e. GSH overproducing tobacco lines) indeed restored wild type levels of TMV resistance in NahG tobacco. Furthermore, overproduction of endogenous glutathione significantly improved TMV resistance regardless whether or not the plants accumulate wild type levels of SA (**Figure 3**).

The above results point to the instrumental role of GSH in conferring defense responses to TMV. Earlier research showed that CEMK-9 and TRI-2 tobacco indeed display significantly elevated GSH levels (Liszewska et al., 2001; Sirko et al., 2004; Wawrzyński et al., 2006), which was confirmed by this study (**Figure 4**) and could explain the enhanced resistance to TMV. In order to clarify if elevated levels of glutathione are indeed maintained in F_1_ hybrids derived from these GSH overproducers (CEMK-9 X Xanthi, TRI-2 X Xanthi, CEMK-9 X NahG, and TRI-2 X NahG), we analyzed levels of total glutathione (GSH and GSSG) in uninoculated, control inoculated (mock), and TMV-inoculated leaves. F_1_ progenies displayed lower foliar glutathione levels than GSH overproducer parental lines (CEMK-9 and TRI-2) but significantly higher levels than in parental Xanthi or NahG. Importantly, high levels of endogenous total glutathione were always associated with enhanced resistance to TMV. Remarkably, however, GSSG levels were unexpectedly high (approximately 50% of total glutathione) in CEMK-9 X NahG and TRI-2 X NahG F_1_ hybrids 5 days after TMV-inoculation (**Figure 4**). It is known that GSSG is reduced by glutathione reductase (GR) to replenish the GSH pool and GR activity and expression of the coding genes are activated by SA (Fodor et al., 1997; Király et al., 2002; Mhamdi et al., 2010). In addition, the GSH/GSSG ratio is dramatically decreased within the first 4 days of TMV infection in SA-deficient NahG tobacco (Király et al., 2002; **Figure 4**), suggesting that the regeneration of GSH is severely impeded in TMV-infected F_1_ progenies of NahG crossed with GSH overproducer tobaccos. The extraordinarily high levels of GSSG in these plants could indicate the pivotal role of glutathione in restoring TMV resistance in the background of SA deficiency, including suppression of oxidative stress (HR) in virus-infected tissues and regulating additional downstream defense responses. For example, it has been shown that an increase in GSSG levels is required for the activation of defense signaling in response to infections by powdery mildews and bacteria (Vanacker et al., 2000; Kovacs et al., 2015).

The fact that GSH overproduction restores wild type levels of TMV resistance in SA-deficient NahG tobacco implies that GSH and SA may induce partially overlapping defense pathways and high levels of GSH may confer TMV resistance by enhancing normal SA accumulation in wild type plants. A significant increase in free and bound SA was evident in TMV-inoculated leaves of wild type and GSH overproducer tobacco lines as compared to controls. Notably GSH overproduction was clearly associated with the highest levels of SA, except for F_1_ hybrids that express *nahG* (CEMK-9 X NahG and TRI-2 X NahG F_1_) (**Figure 5**). Therefore, overexpression of genes involved in cysteine and GSH biosynthesis results not only in GSH overproduction and enhanced TMV resistance but also in elevated levels of SA, indicating that GSH may confer virus resistance in tobacco *via* SA. This is plausible in light of previous reports showing that enhancing GSH biosynthesis by overexpression of *GSH1* in tobacco led to markedly increased SA levels and elevated resistance to pathogens like *Pseudomonas syringae* pv. *tabaci* and *Botrytis cinerea* (Ghanta et al., 2011, Ghanta et al., 2014). Our results demonstrated that overproduction of GSH confers SA accumulation and elevated resistance also to virus pathogens like TMV.

We found that the overproduction of endogenous GSH significantly improved TMV resistance detectable even 5 DAI. To assess the possible role of SA-mediated and GSH-associated defense gene expression in antiviral responses at this advanced stage of TMV pathogenesis we monitored transcript induction of two pathogenesis-related and two GST genes (*NtPR-1a*, *NtPRB-1b, NtGSTtau, NtGSTphi*). Defense gene expressions were significantly higher in TMV-inoculated leaves as compared to controls. However, the highest levels of *PR* and *GST* gene expression were associated with SA-deficiency, rather than enhanced TMV resistance (**Figures 6** and **7**), a possible sign of excessive oxidative stress in virus-infected SA-deficient tobacco cells in later stages of pathogenesis, when HR is fully developed. Previously it has been shown that enhanced HR-type necrotization in TMV-infected NahG tobacco is indeed correlated to oxidative stress, as marked by the accumulation of ROS (Király et al., 2002). In case of SA-deficient F_1_ hybrids that express *nahG* (CEMK-9 X NahG and TRI-2 X NahG F_1_), wild type levels of TMV resistance (including HR necrosis) are coupled to hyperaccumulation of GSSG, an indication of constant suppression of oxidative stress in virus-infected plant cells. The fact that TMV-infected tobacco lines expressing *nahG* display elevated induction of *PR-1* genes at 5 DAI is in line with recent results, showing enhanced expression of *NtPR-1b* and *NtPR-1c* in NahG tobacco in later stages of TMV infection (7 DAI) as compared to wild type (Gullner et al., 2017a). A similarly late expression of *AtPR-1* was also shown in an SA-deficient NahG biotype of *A. thaliana* following infection by an incompatible race of the downy mildew *Hyaloperonospora parasitica* (Delaney et al., 1994). These results imply that although enhanced *PR* gene expression is a marker for SA-mediated resistance in early stages of e.g. viral pathogenesis (van Loon et al., 2006; Király et al., 2012; Gullner et al., 2017a; Klessig et al., 2018), in SA-deficient plants it is clearly a marker of host stress and/or a failed resistance response in advanced stages of infection. We also showed that TMV-infected tobacco lines expressing *nahG* display elevated induction of two *GST* genes (*NtGSTtau, NtGSTphi*) at 5 DAI, an additional marker of host stress and impaired resistance to TMV. Indeed, it has been reported that two *GST* genes are upregulated in pepper resistant to *Capsicum chlorosis virus* (CaCV) at the time point when HR-type lesions are fully developed (Widana Gamage et al., 2016). Also, inoculation of potato with *Potato virus Y* (PVY) NTN that causes severe chlorotic/necrotic ringspots results in elevated expression of a *GST*, as compared to infections by the mild N strain (Kogovsek et al., 2010). In potato leaves systemically infected with PVX, the appearance of mosaic symptoms was associated with overexpression of *GST* and *PR-1* genes (Niehl et al., 2006). However, a lack of correlation between virus titers and defense gene expression suggests that these plant responses are primarily directed against oxidative stress, rather than against the invading virus.

Previous studies showed that enhancing GSH biosynthesis leads to improved resistance to bacterial and fungal pathogens *via* elevated SA levels (Ghanta et al., 2011, Ghanta et al., 2014). However, our results demonstrated that GSH overproduction confers SA accumulation and elevated resistance also to a virus pathogen, TMV. Furthermore, we have shown that in TMV-infected tobacco GSH may even compensate for SA deficiency to maintain virus resistance: elevation of endogenous GSH levels in SA-deficient NahG tobacco, both genetically and by exogenous treatments, restores wild type levels of TMV resistance. These findings underline the importance of glutathione-induced redox changes in plant cells during signaling processes leading to antiviral defense reactions. We suppose that elevated levels of GSH enable virus-infected plants not only to maintain a reduced environment but also conferring a long lasting resistance to the invading pathogen.

## Data Availability

All datasets generated for this study are included in the manuscript and the **Supplementary Files**.

## Author Contributions

AE, AK, GG, and LK conceived the idea and wrote the manuscript. AK and LK carried out the molecular biological experiments, while AK, GG, and KG made the chromatographic analyses.

## Funding

The financial support of the Hungarian National Research, Development and Innovation Office (NKFIH PD-108455, K-124131 and K-128868) and of the Hungarian-American Fulbright Commission for Educational Exchange for GG is gratefully acknowledged.

## Conflict of Interest Statement

The authors declare that the research was conducted in the absence of any commercial or financial relationships that could be construed as a potential conflict of interest.
