# Supplementary material for: Glutathione Can Compensate for Salicylic Acid Deficiency in Tobacco to Maintain Resistance to Tobacco Mosaic Virus
Source: Front Plant Sci. 2019 Sep 13;10:1115. doi: 10.3389/fpls.2019.01115 (PMC6769422; doi:10.3389/fpls.2019.01115)
Supplement: Supplementary file 1 [file Table_1.docx]

**SUPPLEMENTAL FIGURES**

**FIGURE S1**

Changes of free and bound salicylic acid (SA) contents in leaf discs (diameter 1.5 cm) of tobacco (*Nicotiana tabacum* cv. Xanthi NN) plants floating on the surface of 2 mM or 5 mM aqueous solutions of R-2-oxo-4-thiazolidine-carboxylic acid (OTC) or water for 1, 2 and 3 days in Petri dishes as described by Gullner et al. (1999). Incubations were carried out in a growth chamber under a 16/8 hrs light/dark regime at 22 °C. Free and bound SA levels were determined by HPLC and spectrofluorometric detection as described by Enyedi et al. (1992). Means of three independent experiments ± SD are shown. The symbols *, ** and *** show significant differences at P < 5%, P < 1% and P < 0.1%, respectively, as determined by Student's t-test. OTC treatments were compared to their corresponding water controls in a pairwise manner for each day, separately.

**FIGURE S2**

Changes of free and bound salicylic acid (SA) contents in leaf discs (diameter 1.5 cm) of Xanthi NN and NahG tobaccos floating on the surface of a 2 mM aqueous solution of R-2-oxo-4-thiazolidine-carboxylic acid (OTC) or water for 2 days as described by Gullner et al. (1999). Incubations were carried out in a growth chamber under a 16/8 hrs light/dark regime at 22 °C. Free and bound SA levels were determined by HPLC and spectrofluorometric detection as described by Enyedi et al. (1992). Means of three independent experiments ± SD are shown. The symbols * and *** show significant differences between OTC-treated and water-treated (control) leaves at P < 5% and P < 0.1%, respectively, as determined by Student's t-test.


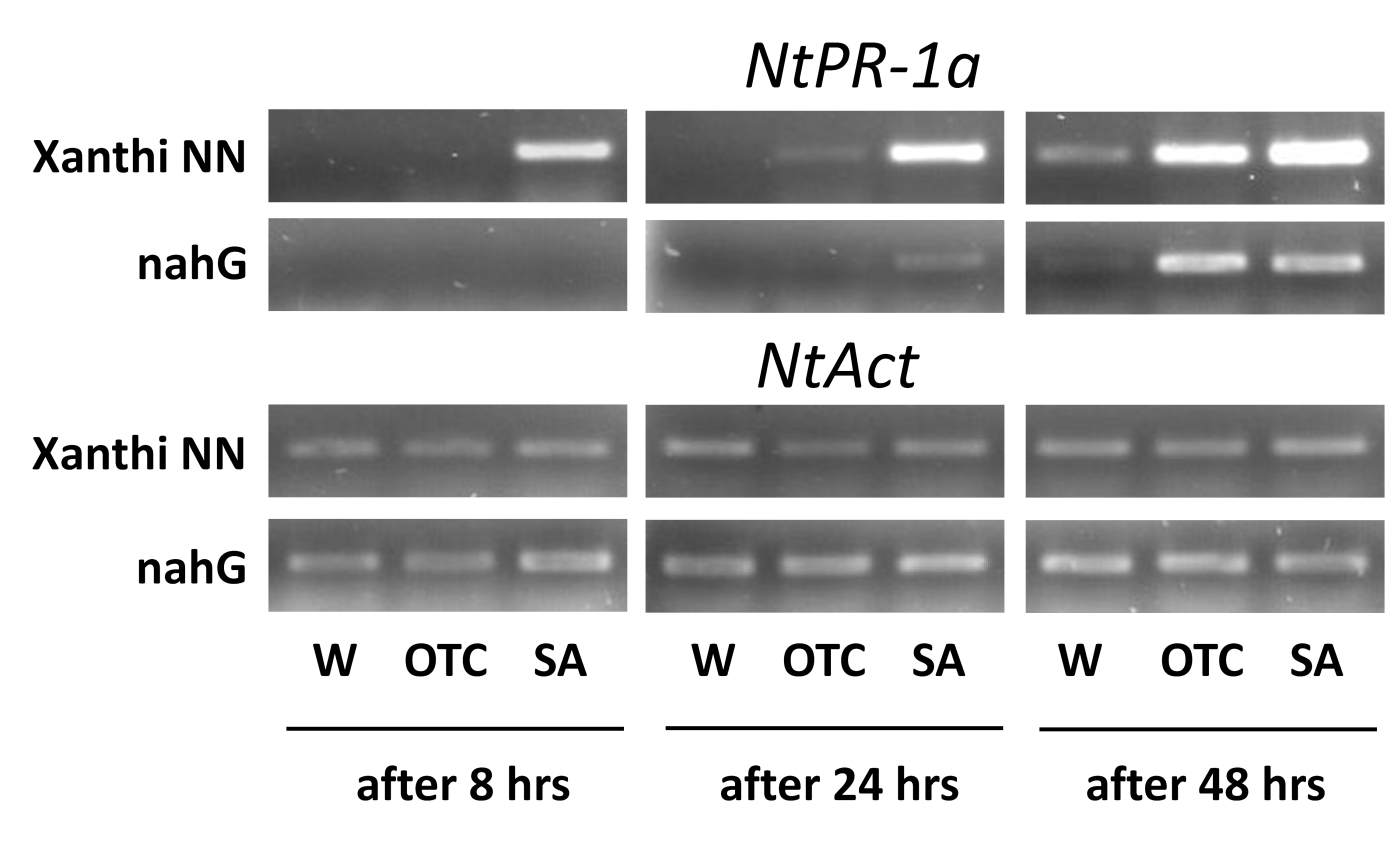


**FIGURE S3**

Up-regulation of expression of the *NtPR-1a* gene in leaf discs of Xanthi NN and NahG tobaccos floating on aqueous solutions of salicylic acid (SA, 0.2 mM), R-2-oxo-4-thiazolidine-carboxylic acid (OTC, 2 mM) or water (W) for 8, 24 and 48 hours as described by Gullner et al. (1999). Gene expression levels were determined by semiquantitative RT-PCR using expression of an actin gene (*NtAct*) as a reference according to Höller et al. (2010). Representative results of two independent biological experiments are shown.
